# Preparation and Characterization of Zn(II)-Stabilized Aβ_42_ Oligomers

**DOI:** 10.1021/acschemneuro.4c00084

**Published:** 2024-07-09

**Authors:** Alicia González Díaz, Rodrigo Cataldi, Benedetta Mannini, Michele Vendruscolo

**Affiliations:** † Centre for Misfolding Diseases, Yusuf Hamied Department of Chemistry, University of Cambridge, Cambridge CB2 1EW, United Kingdom; ‡ Department of Experimental and Clinical Biomedical Sciences Mario Serio, University of Florence, 50134 Florence, Italy

**Keywords:** Aβ42, oligomers, zinc, Alzheimer′s disease, neurodegeneration

## Abstract

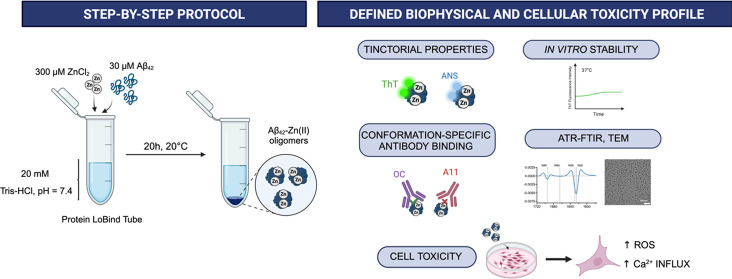

Aβ oligomers are being investigated as cytotoxic agents in Alzheimer’s disease (AD). Because of their transient nature and conformational heterogeneity, the relationship between the structure and activity of these oligomers is still poorly understood. Hence, methods for stabilizing Aβ oligomeric species relevant to AD are needed to uncover the structural determinants of their cytotoxicity. Here, we build on the observation that metal ions and metabolites have been shown to interact with Aβ, influencing its aggregation and stabilizing its oligomeric species. We thus developed a method that uses zinc ions, Zn(II), to stabilize oligomers produced by the 42-residue form of Aβ (Aβ_42_), which is dysregulated in AD. These Aβ_42_-Zn(II) oligomers are small in size, spanning the 10–30 nm range, stable at physiological temperature, and with a broad toxic profile in human neuroblastoma cells. These oligomers offer a tool to study the mechanisms of toxicity of Aβ oligomers in cellular and animal AD models.

## Introduction

Alzheimer′s disease (AD) is the most common cause of dementia, a condition affecting over 50 million patients and representing a major global socioeconomic problem.^1,2^ Although the molecular events underlying the onset and progression of AD remain poorly understood, an imbalance between Aβ production and degradation pathways, leading to Aβ accumulation and aggregation, is thought to play a central role.^3,4^ Upon abnormal processing of the amyloid precursor protein (APP), Aβ peptides of different lengths are generated, with Aβ_40_ and Aβ_42_ being the prevalent forms.^3,4^ While both peptides are main components of amyloid plaques, Aβ_42_ is more amyloidogenic and neurotoxic.^3,4^

Misfolded Aβ monomers can assemble in oligomeric species, which have been implicated in the pathogenesis of AD.^5,6^ For this reason, Aβ oligomers have been intensively pursued as targets for drug discovery.^7−11^ These efforts would be facilitated by a detailed knowledge of the structural properties of these targets. However, oligomeric species encompass a wide variety of metastable misfolded assemblies, with a transient and heterogeneous nature that makes their isolation and characterization a challenging task.^12−14^ As a consequence, although a variety of methods have been proposed and utilized for systematic studies of the properties of Aβ oligomers, there is still a lack of consensus about the identity and properties of the Aβ oligomers that are relevant in AD. Some approaches generate stable Aβ-derived diffusible ligands (ADDLS) or protofibrils using chemically synthesized peptides.^15,16^ Other approaches apply protein engineering to induce the formation of disulfide-cross-linked dimers.^17^ Aβ oligomers have been directly extracted from homogenates of postmortem AD samples,^18−21^ while other studies have described the isolation of Aβ monomers and small oligomers from the supernatant of cells in culture, such as 7PA2.^22^ However, it remains difficult to establish whether the aggregation state of those protein species is altered during the isolation process, and thus, the studied biophysical properties are representative of the original aggregates.

A strategy to overcome the problem of identifying the types of Aβ oligomers relevant in AD involves the compilation of a panel of well-characterized Aβ oligomers that collectively recapitulate the properties of those present in AD brains.^23,24^ Toward this challenging goal, the present study was inspired by the inorganic chemistry aspects of AD, which have caught the attention of researchers in recent years.^25−27^ Postmortem examination of AD brains suggested that metal ions such as Cu(II), Fe(III), and Zn(II), found in high concentrations within the core and periphery of senile plaques, play a role in modulating the dynamics of Aβ aggregation.^28,29^ In fact, Aβ, as well as many amyloid-forming proteins, can interact with these transition-metal ions, which causes changes to their structure and function.^30,31^ In particular, the interaction of Aβ with Zn(II) has been extensively characterized in several *in vitro* studies.^32−34^ A Zn(II) binding site for Aβ is located in the N-terminus (Asp1–Lys16).^32^ A related binding model was determined by the use of NMR spectroscopy, where Zn(II) was found to have a tetrahedral coordination environment with the involvement of three histidine residues (His-6, His-13, and His-14).^33^ More recent simulation studies also revealed that Zn(II) can bind to Glu22 on the Aβ16–22 peptide and disrupt the formation of salt bridges during the assembly of this fragment, which are pivotal for stabilizing antiparallel β-sheet structures.^35^ In the presence of Zn(II), the authors showed a decrease in the probability of the formation of antiparallel β-sheets favoring the formation of parallel β-sheet structures instead. This study underscores the impact of Zn(II) on Aβ assembly and demonstrates that the manipulation of Zn(II) levels could potentially influence the structural configuration of amyloid aggregates. The chelation of Aβ with Zn(II) has also been reported to affect its aggregation behavior. A recent investigation described the influence of stoichiometric Zn(II) on the aggregation kinetics of Aβ_42_ and showed that, under certain experimental conditions, Zn(II) can favor the generation of off-pathway aggregates, with reduced β-sheet structures.^36^

In a physiological context, Zn(II) plays a crucial role in brain functions such as neurotransmission and synaptic plasticity.^37,42^ Research on Zn(II) levels in AD patients, however, presents conflicting results.^37−43^ Elevated Zn(II) has been reported in brain regions with significant amyloid deposition,^38^ while decreased levels have been observed in other AD brain areas and patients’ serum, correlating with exacerbated cognitive decline.^39−41^ One theory to explain these observations suggests that physiological Zn(II) concentrations, which can reach up to 300 μM in the extracellular space of brain synapses, might bind to Aβ peptides, promoting Aβ oligomerization and aggregation in amyloid plaques.^39^ As a result, Zn(II) captured by these plaques becomes less available for essential brain functions, potentially worsening cognitive function and neurodegeneration.

Despite all of this evidence, there is still a need to develop standardized methods that use Zn (II), among other brain-relevant metal ions, to stabilize Aβ oligomeric species that may mimic those found in the AD brain. These stabilized oligomers hold the potential to serve as valuable tools in deciphering the structural determinants contributing to cytotoxicity of Aβ aggregates, thereby facilitating the screening and development of novel AD therapeutics. In this work, we report a method to prepare and characterize Zn(II)-stabilized Aβ_42_ oligomers taking advantage of previous literature on Zn(II)-stabilized Aβ_40_ oligomers.^23^ Although Zn(II)-induced Aβ_42_ oligomer formation has been previously reported,^36,44−47,75,75A^ to our knowledge there is still a need for standardized, step-by-step protocols, such as the one reported here, to enable the production of stable Zn(II)-stabilized Aβ_42_ oligomers with well-characterized biophysical and cell toxicity properties.

## Results

### Effects of Zn(II), Cu(II), and DOPAL on Aβ_42_ Aggregation Kinetics

**Figure 1:**
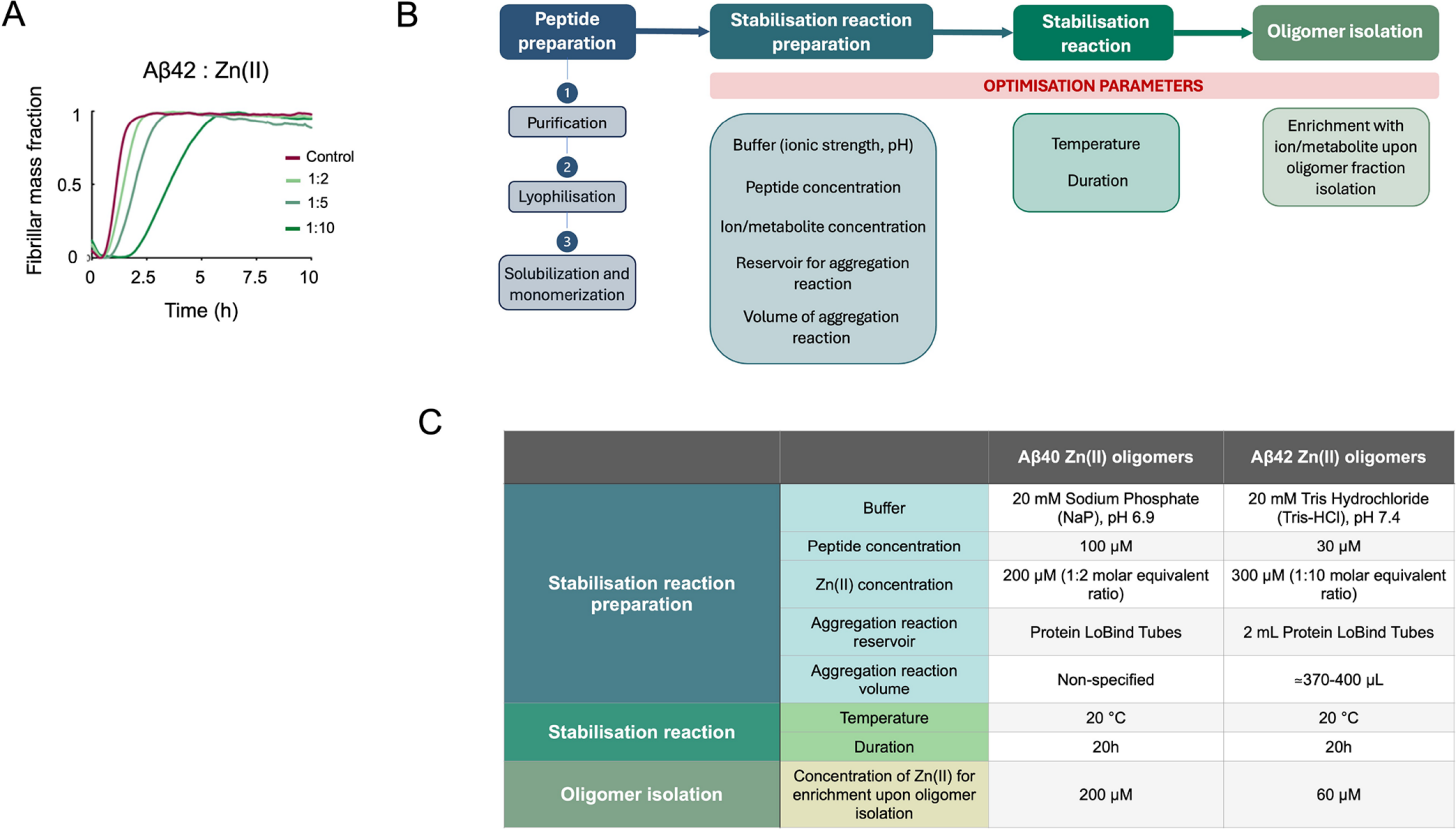
Using Zn(II) for the optimization of a protocol to stabilize Aβ_42_ oligomers: (A) ThT fluorescence profiles of the aggregation of freshly purified monomeric Aβ_42_ at 5 μM in the absence or presence of increasing molar equivalents of Zn(II). (B) Scheme illustrating the main steps of a previously reported stabilization protocol.^23^ We propose an optimization workflow to apply this protocol for the stabilization of aggregation-prone peptides, such as Aβ, by fine-tuning key experimental variables related with (i) the preparation of the stabilization reaction; (ii) the environmental conditions for the stabilization reaction; and (iii) the isolation and manipulation of the generated oligomeric species. (C) Experimental modifications of the Aβ_40_-Zn (II) protocol to generate stable Aβ_42_-Zn(II) oligomeric species.

We first aimed to assess whether Zn(II) ions, together with a different metal ion (Cu(II)) and one metabolite (3,4-dihydroxyphenylacetaldehyde, DOPAL)—reported to bind Aβ peptides—were able to interfere with the aggregation of monomeric Aβ_42_ (Figures 1A and S1A–B). The aggregation process was monitored using the amyloid-binding dye thioflavin T (ThT), whose fluorescence increases upon interaction with amyloid structures.^48^ As previously shown for Aβ_40_, Zn(II) is able to inhibit the aggregation of Aβ_42_ in a dose-dependent manner (Figure 1A). Similar results were observed for Cu(II), while only an increase in the *t*_1/2_ was noticed for the highest tested molar equivalent ratio of Aβ_42_:DOPAL (1:10) (Figure S1).

Previous reports showed that high concentrations of Zn(II), Cu(II), and DOPAL can inhibit Aβ aggregation by promoting the formation of off-pathway aggregates.^23,24^ However, we note that different experimental conditions such as pH, ionic strength, or protein concentration can lead to the generation of Aβ-derived species with heterogeneous morphologies and aggregation behaviors.^23,24^ In the present study, we aim to standardize a method to generate to generate homogeneous population of stable Aβ_42_ oligomers amenable to biophysical characterization, taking advantage of the ability of Zn(II) to interact with monomeric Aβ_42_. In parallel, we present our method as a workflow for protocol optimization (Figure 1B) to further explore the potential of Cu(II) and DOPAL, among other relevant Aβ aggregation modulators, to stabilize other forms of Aβ_42_ oligomers.

### Protocol to Stabilize Aβ_42_ Oligomers Using Zn(II) Ions

A protocol for the stabilization of Aβ_40_ oligomers using Zn(II) was recently reported.^23^ We adopted that procedure here as the starting point for the preparation of Aβ_42_ oligomers. The protocol is composed by three main steps: (i) monomerization of the Aβ_42_ peptide and preparation of the stabilization reaction; (ii) incubation of monomeric Aβ_42_ with Zn(II) (stabilization reaction); and (iii) isolation and characterization of the generated oligomers (Figure 1B).

Because of the higher aggregation propensity of Aβ_42_ as compared to Aβ_40_, several variations were introduced to the reference protocol^23^ (Figure 1C). (i) The protein concentration was decreased from 100 μM (reported for Aβ_40_) to 30 μM to maximize oligomer stability and yield. (ii) 20 mM sodium phosphate buffer, commonly used for Aβ purification, aggregation kinetics, and Aβ_40_ Zn(II)-oligomer preparations, was replaced by a buffer with lower ionic strength (20 mM Tris hydrochloride) for the stabilization reaction. By doing so, we aimed to prevent free phosphate groups to form complexes with zinc ions, decreasing their availability to interact with Aβ_42_ peptides and hence reducing the generation of stable, off-pathway aggregates. (iii) To further increase the levels of Zn(II) available to interact with the Aβ_42_ peptides, we increased their molar excess from 2:1 to 10:1 with respect to the monomer. (iv) Moreover, since the effect of the air–water interface has been reported to influence the kinetics of protein aggregation,^49^ to minimize variability among oligomer preparations, the stabilization reaction was always carried out in a final volume of 370 μL in 2 mL Protein LoBind tubes. (v) Finally, after isolation of oligomer species by centrifugation of the stabilization reaction product, oligomers were resuspended in Tris-HCl buffer enriched with 60 μM Zn(II) to improve stability.

### Biophysical Features of the Time Course of the Stabilization Reaction

Following this protocol, Aβ_42_ monomers were incubated in the presence of Zn(II) at 20 °C for 10 min and 4, 8, and 20 h before the aggregates were isolated by centrifugation and resuspension. A control reaction without Zn(II) was always run in parallel. The species generated at each time point were structurally characterized using biophysical techniques to assess which incubation time yielded stable oligomeric species.

**Figure 2:**
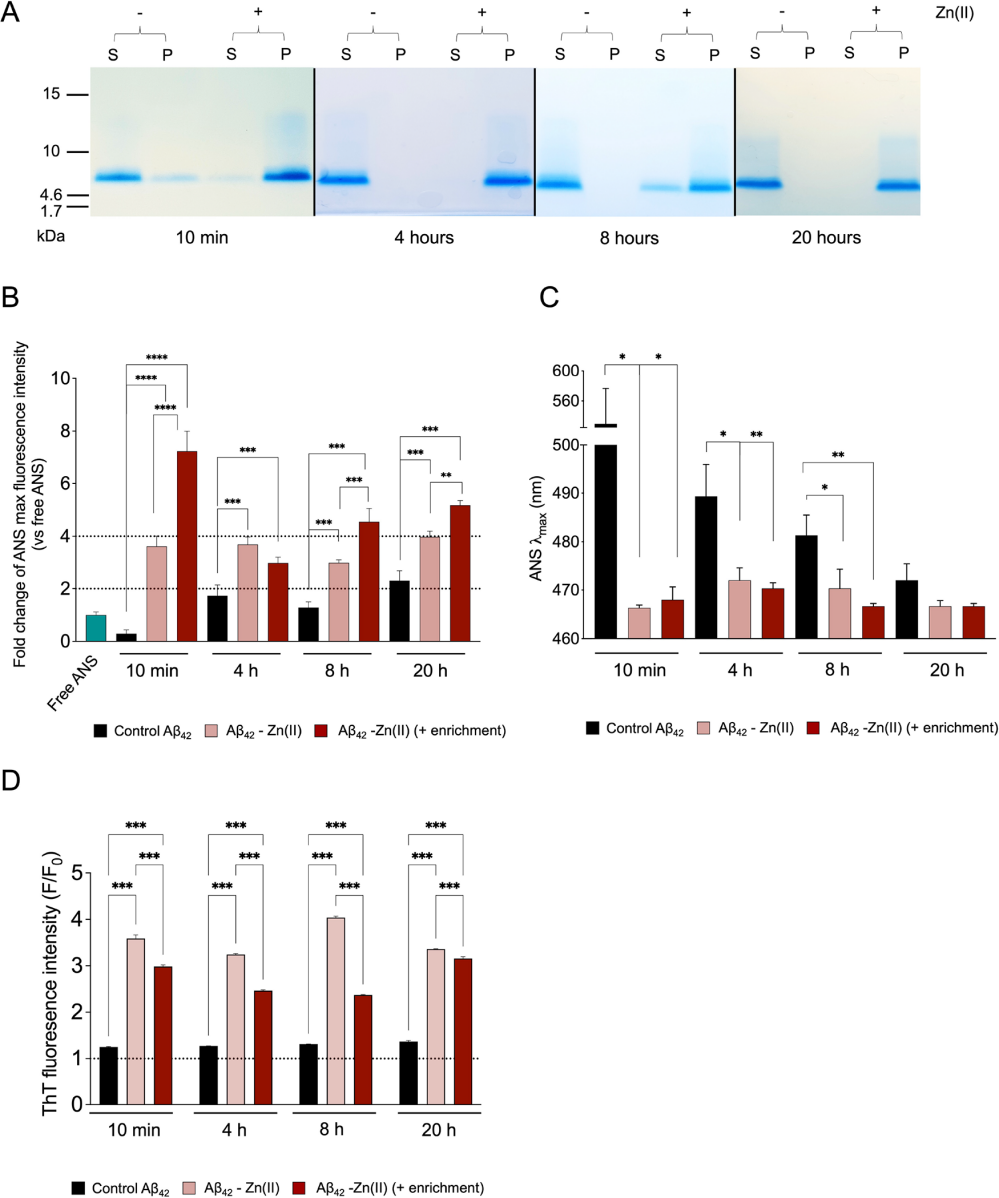
Biophysical features of the reaction of stabilization of Aβ_42_ oligomers by Zn(II). (A) SDS-PAGE representing protein species distribution in supernatant (S) and pellet (P) fractions of stabilization reactions run for 10 min, 4, 8 and 20 h at 20 °C in the absence (−) or presence (+) of 1:10 molar excess of Zn(II). (B,C) Fold change of ANS-derived maximum fluorescence intensity calculated over free ANS dye and maximum ANS emission wavelength (λ_max_) of stabilization reaction products run in the absence (control Aβ_42_) or presence of Zn(II) with or without further Zn(II) enrichment of the pellet fraction (Aβ_42_-Zn(II)). (D) F/F_0_ ratio between the ThT fluorescence intensity at 485 nm in the presence (F) and absence (Fo) of protein of the supernatant fraction of the control sample and the pellet fractions of enriched and nonenriched Aβ_42_-Zn(II) samples after 10 min, 4, 8, and 20 h of stabilization reaction. Error bars represent standard deviation. Statistical analysis was performed by a one-way ANOVA within each time point, applying Bonferroni correction for multiple comparisons. *p-value < 0.1, **p-value < 0.01, and ***p-value < 0.001.

We first assessed the distribution of Aβ_42_ species and their relative concentrations in the supernatant and pellet fractions after each stabilization reaction (10 min, 4 h, 8 h, and 20 h at 20 °C). The SDS-PAGE assay revealed that, in the absence of Zn(II), after 10 min of incubation at 20 °C, around 80–90% of the total Aβ_42_ species remained in the supernatant of the control reactions, while 10–20% of the aggregates were found in the pellet fraction (Figure 2A). Sample distribution was reversed when the aggregation occurred in the presence of Zn(II). Notably, with the progression of the reaction, 100% of the protein species were found in the supernatant of control reactions and in the pellet of Zn(II) reactions, respectively. This difference was presumably due to alternative physicochemical properties of the generated aggregates. Over the course of the aggregation reaction, processes of monomer association and dissociation into and from Zn(II)–protein complexes are expected to occur until full conversion into stable Zn(II) oligomers. These dynamic changes in the nature of the aggregates generated over time may explain why observed soluble species appear and disappear from the supernatant fraction of the Zn(II) samples across the explored time points.

Aβ_42_-enriched fractions were then subjected to biophysical characterization in bulk as initial test of the reactions that potentially generated oligomeric-like species. Misfolding and aggregation of Aβ_42_ give rise to small, highly hydrophobic oligomers with low levels of β-sheet content,^50^ which then convert into bigger-sized aggregates such as protofibrils and fibrils, rich in the canonical cross-β structure and with lower extent of exposed hydrophobic regions.^50^ In this context, three bulk biophysical assays were performed to estimate the average hydrophobicity (ANS assay), cross-β content (ThT assay), and size (turbidimetry assay) of the generated Aβ_42_ aggregates.

ANS is a fluorescent probe able to bind to hydrophobic residues exposed on the surface of misfolded proteins.^51^ Upon binding, the fluorophore exhibits an increase in the fluorescence emission intensity and a blue shift of the maximum emission wavelength (λ_max_).

ANS fluorescence emission spectra were obtained from control Aβ_42_ and Aβ_42_-Zn(II) samples with and without further Zn(II) enrichment after incubation at 20 °C for 10 min, 4 h, 8 h, and 20 h. The maximum ANS fluorescence intensity (ANS_max_) was normalized with respect to the fluorescence derived from the free ANS dye in solution for each time point (Figure 2B,C).

Just after 10 min of stabilization reaction, highly hydrophobic species were generated in the presence of Zn(II). In particular, ANS_max_ measurements showed that Aβ_42_-Zn(II) reaction products that were not enriched with Zn(II) after resuspension yielded species that, on average, exposed four times more hydrophobic patches as compared to controls (Figure 2B). This fold change doubled upon Zn(II) enrichment. Notably, ANS differences between control and Aβ_42_-Zn(II) reaction products were preserved for all later time points but became less prominent due to the evolution of the aggregated state of both control and Aβ_42_-Zn(II) reaction products (Figure 2B).

Beyond 4 h of stabilization reaction, ANS_max_ of enriched Aβ_42_-Zn(II) samples decreased as compared to 10 min reaction products while still being higher than the control reaction products. ANS_max_ mean values of enriched Aβ_42_-Zn(II) samples kept increasing over time (fold change (FC) vs control ≃3 at 4 h; ≃4.5 at 8 h, and ≃5 at 20 h), On the contrary, the ANS_max_ value of nonenriched Aβ_42_-Zn(II) reaction products did not show significant variation between any explored time point (FC vs control fluctuates ≃3–4 along the stabilization reaction).

The increase in ANS_max_ in the Zn(II) reaction products was accompanied by a shift to lower λ_max_. After 10 min of reaction, the bigger difference between control and Aβ_42_-Zn(II) samples λ_max_ was observed (Aβ_42_-Zn(II) λ_max_ ≃ 465–470 nm, Aβ_42_-control λ_max_ ≃ 520 nm, *p*-value < 0.05). However, these λ_max_ differences got less pronounced as the aggregation reaction progressed over time due to the fact that hydrophobic species were generated also in the absence of Zn(II). Additionally, λ_max_ values of both enriched and nonenriched Aβ_42_-Zn(II) samples fluctuated within a small range of values (≃465–472) independently of the time point (Figure 2C).

Taken together, these results indicate an increase in the mean hydrophobicity levels of Zn(II) samples as compared to the control, with a more apparent effect upon Zn(II) enrichment after the stabilization reaction.

#### ThT-Binding Assay

For every explored time point, we then performed ThT end point assays to assess the average cross-β formation of the generated species right after each stabilization reaction and ThT kinetics assays to monitor the stability of the aggregates by following the changes in their cross-β levels over time.

End point ThT assays showed that control reactions, run in the absence of Zn(II), did not demonstrate an increase in the ThT-derived F/F_0_ signal upon increasing incubation times. However, when the aggregation reaction happened in the presence of Zn (II), ThT fluorescence intensity appeared to be significantly higher than the control, already after 10 min of incubation and during the whole duration of the stabilization reaction, in line with the ANS results (Figure 2C,D). Notably, Zn(II) enrichment significantly decreased the mean cross-β content of the generated species, independently of the time point.

Taken together, these results suggest that only after 10 min of incubation at 20 °C, Aβ_42_ aggregated in the presence of Zn(II)-generated species competent to bind to ThT and ANS dyes. The mean hydrophobicity and β-sheet content levels fluctuated along the stabilization reaction time course. This implies generated protein populations arrange dynamically into different conformations over time. Furthermore, enrichment with Zn(II) consistently increased the ANS and decreased the ThT signal as compared to nonenriched samples, indicating that the levels of free Zn(II) in the buffer seemed to further alter the structural properties of the generated aggregates.

**Figure 3:**
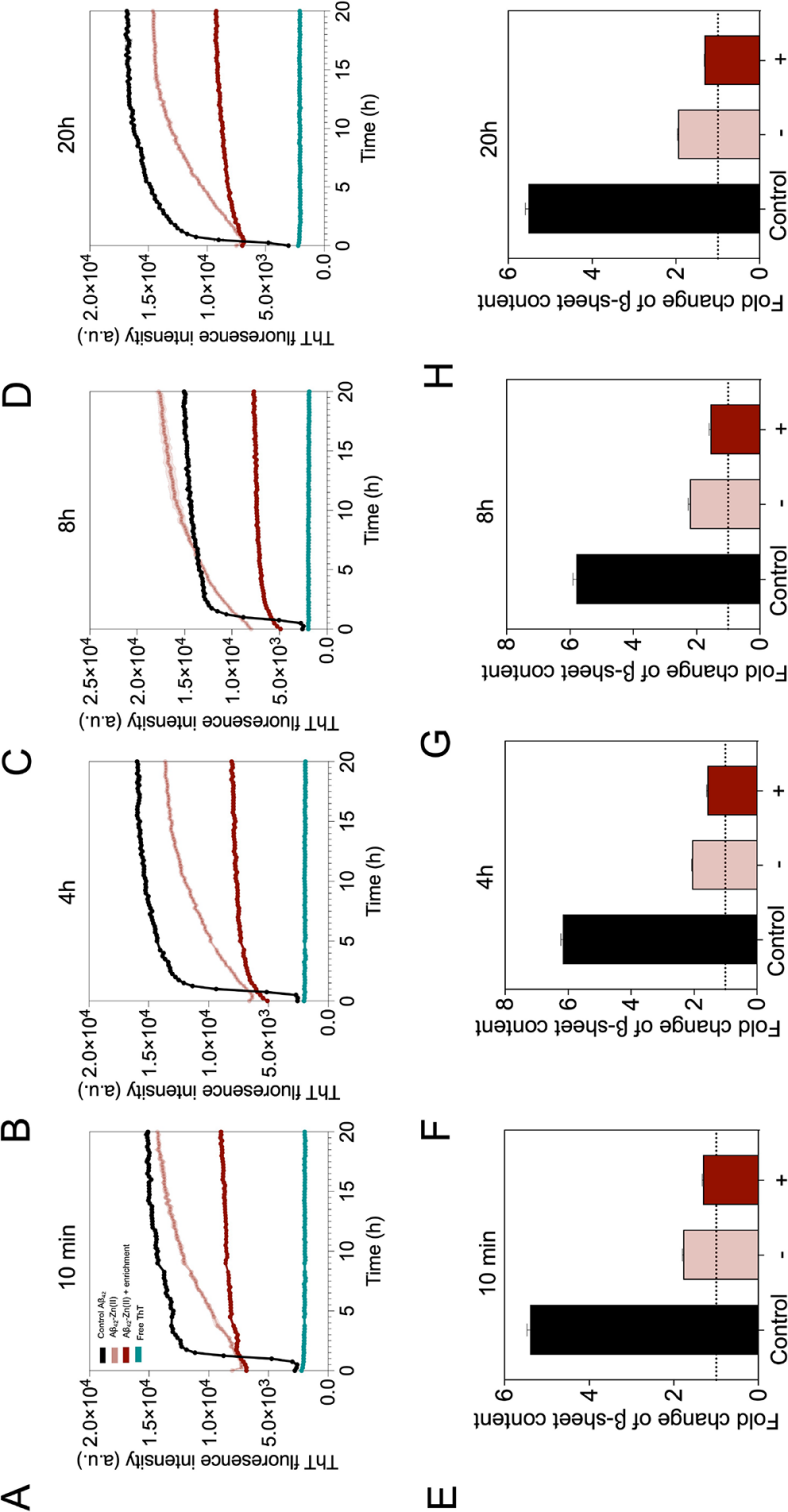
Stability of Aβ_42_ species generated over the stabilization reaction time course. Raw ThT fluorescence data representing the progression of cross-β formation over a total of 20 h of aggregation at 37 °C for the control Aβ_42_ and enriched and nonenriched Aβ_42_-Zn(II) samples after (A) 10 min, (B) 4 h, (C) 8 h, and (D) 20 h of stabilization reaction at 20 °C. Fold change of average cross-β formation of control Aβ_42_, nonenriched (−), and enriched (+) Aβ_42_-Zn(II) samples incubated for 20 h at 37 °C after (E) 10 min, (F) 4 h, (G) 8 h, and (H) 20 h of stabilization reaction.

In another set of experiments, we then analyzed the stability of the aggregated species at physiological temperature (37 °C) after being subjected to 10 min, 4 h, 8 h, and 20 h of stabilization reaction (Figure 3). For that purpose, ThT kinetics raw data were used to establish the fold change of cross-β content of each time point of our stabilization reaction, calculated as the ThT fluorescence intensity ratio between the *t*_0_ (0 h) and *t*_plateau_ (20 h) of each kinetic reaction run at 37 °C. Control reaction products, preaggregated for 10 min to 20 h at 20 °C in the absence of Zn(II), suffered a 4- to 6-fold increase in β-sheet secondary structures upon incubation at a physiological temperature (Figure 3). On the contrary, monomers preaggregated with Zn(II) at 20 °C generated species which cross-β content duplicated after 20 h of incubation at 37 °C. Further enrichment with Zn(II) prevented aggregation of the generated species whose amyloid content minimally changed at 37 °C (FC of cross-β content ≃ 1.3 after 20 h of incubation). Mean values of the graphs reported in Figure 3 are summarized in Table S1.

**Figure 4:**
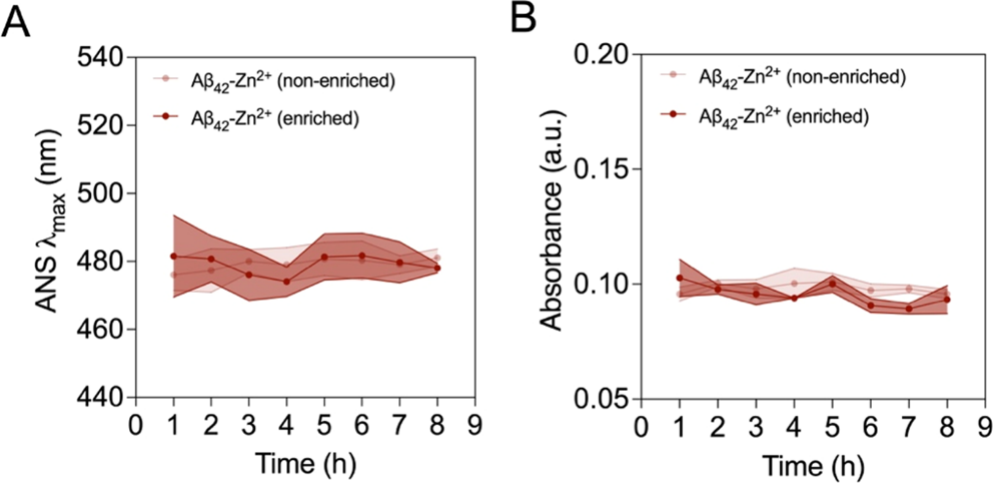
Progression of the mean hydrophobicity and turbidity of stabilized Aβ_42_-Zn(II) species at physiological temperature. (A) Progression of the wavelength of maximum ANS-derived fluorescence emission of both enriched and nonenriched M-Aβ_42_-Zn(II) samples subjected to a stabilization reaction of 20 h at 20 °C and further incubated for 8 h at 37 °C. Error bars represent standard deviation from three technical replicates. (B) Progression of the average size of enriched and nonenriched M-Aβ_42_-Zn(II) samples under the same conditions. No significant differences were observed for both readouts after performing a one-way ANOVA with Bonferroni correction for multiple comparisons.

We then examined whether other relevant bulk biophysical features, such as hydrophobicity and average size of the aggregates, remain unaltered when species stabilized for 20 h were incubated at 37 °C for 8 h. ANS-derived fluorescence was used for bulk hydrophobicity estimation. The average size of the aggregated protein species was assessed by measuring the turbidimetry of the solution from the absorbance of the sample at λ_500_. As shown in Figure 4, no significant changes in ANS λ_max_ and sample turbidity were observed over 8 h of incubation at 37 °C for both Zn(II)-enriched and nonenriched Aβ_42_ samples preaggregated for 20 h at 20 °C.

In summary, by incubating monomeric Aβ_42_ for a total of 20 h at 20 °C, in the presence of 1:10 molar excess of Zn(II) in 20 mM Tris-HCl pH 7.4, together with the addition of Zn(II) upon isolation of the aggregates after the stabilization reaction, we generated species whose biophysical properties measured in bulk are typical of oligomeric species, which are stable when incubated at 37 °C. This improvement in the stability of the oligomers supposed a major headway as it extended the time frame for further characterization of their biophysical and cellular toxicity properties.

### Exploration of the Structures of Zn(II)-Stabilized Aβ_42_ Oligomers

We then set out to conduct a more detailed structural characterization of Aβ_42_-Zn(II) aggregates and control samples by (i) addressing their secondary structures using Fourier transform infrared spectroscopy (FTIR), (ii) assessing their antibody-specific reactivity by dot-blot techniques, and (iii) studying of their morphology and heterogeneity by transmission electron microscopy (TEM).

#### ATR-FTIR and Far-UV Circular Dichroism of Zn(II)-Stabilized Aβ_42_ Oligomers

**Figure 5:**
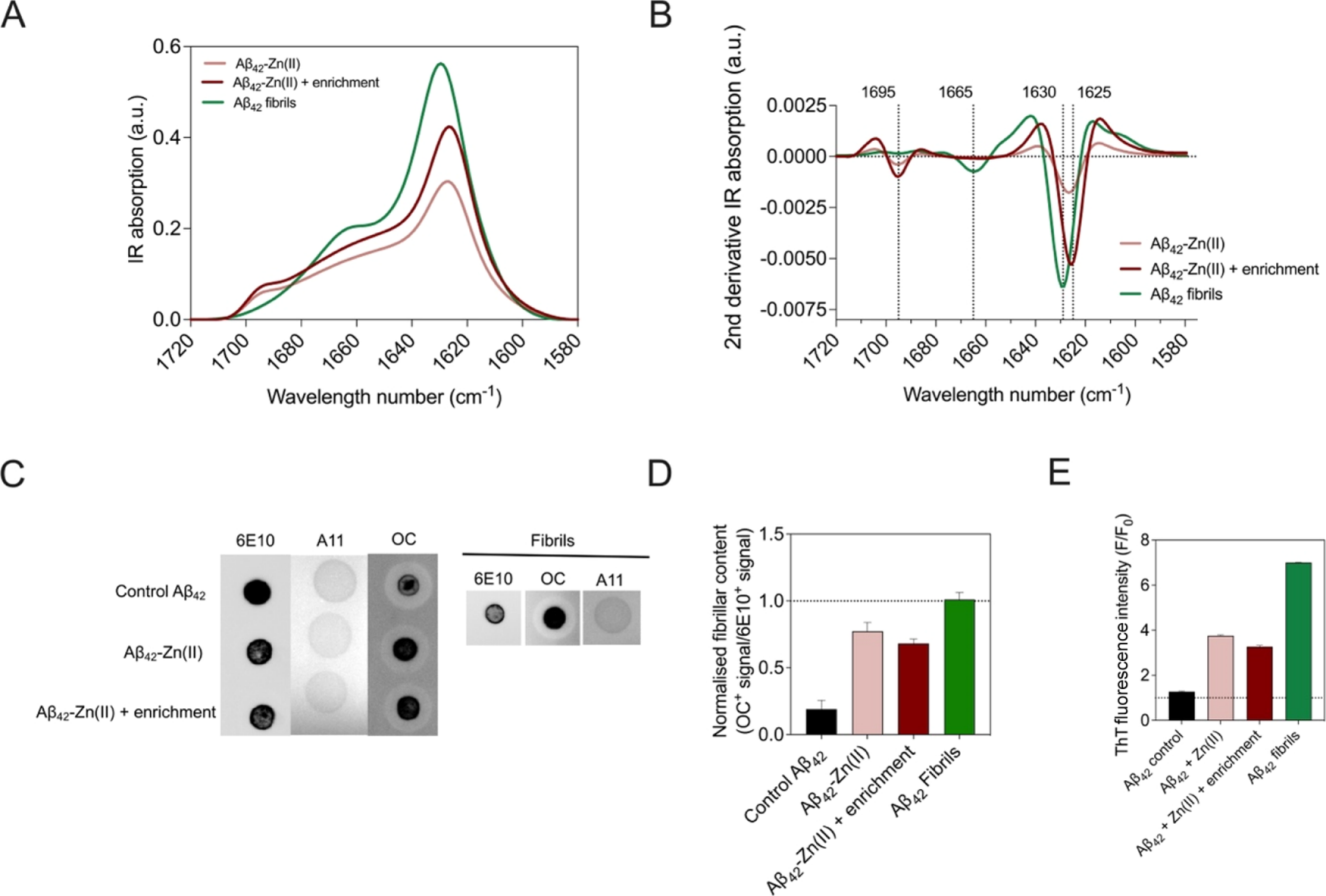
Secondary structures of Aβ_42_-Zn(II) oligomeric species. (A) ATR-FTIR and (B) second derivative of the ATR-FTIR spectrum of Aβ_42_-derived fibrils and enriched and nonenriched Aβ_42_-Zn(II) samples after a stabilization reaction of 20 h at 20 °C. Oligomer-like species displayed significant antiparallel (1695 nm) and parallel (1630–1625 nm) β-sheet structure. (C) Representative dot-blot showing the antibody-specific reactivity of enriched and nonenriched Aβ_42_-Zn(II) samples after 20 h of stabilization reaction at 20 °C, together with Aβ_42_ fibrils. The sequence-specific 6E10 antibody was used as the peptide loading control. A11 and OC were used as conformation-specific antibodies for prefibrillar (A11) or fibrillar (OC) oligomeric or aggregate structures. (D) Total OC-derived signal per sample divided by the total 6E10-derived signal, represented as the normalized amyloid content per sample, showcasing intermediate levels of OC staining for Aβ_42_-Zn(II) samples as compared to the control and fibrils. (E) F/F0 ratio of the ThT fluorescence intensity at 485 nm in the presence (F) and absence (F0) from the four samples tested for their antibody reactivity (Aβ42 control, non-enriched or enriched Aβ42-Zn(II) species and Aβ42 fibrils).

When exposed to a continuum source of infrared (IR) light, proteins can absorb at characteristic wavelengths corresponding to their molecular structures. This absorption causes intramolecular vibrations that can be monitored with an IR detector. It has been described that peptide groups can give rise to 9 IR bands, referred to as A, B, and I–VII bands.^52^ Amide I band specifically carries information on the protein backbone conformation. Methods have been developed for their deconvolution and correlation with the presence and richness of parallel and antiparallel β-sheet structures, α-helix, and random coils or turns within the examined peptides.^53^ As shown in Figure 5A,B, secondary structures of enriched and nonenriched Aβ_42_-Zn(II) samples were comparatively analyzed against Aβ_42_ fibrils. Raw IR spectra (Figure 5A) were deconvoluted and subjected to a second derivative before peak identification. First, Aβ_42_-Zn(II) samples showed a peak at 1695 cm^–1^ wavelength number, totally absent in the case of amyloid fibrils. This peak corresponds to the antiparallel β-sheet structure, and it is a feature reported to be present in other types of amyloid oligomers and absent in fibrils.^54−56^ A negative peak at 1665 cm^–1^ wavelength number, characteristic of an α-helix bend, was present in amyloid fibrils. Finally, from the range of wavelength numbers 1630–1625 cm^–1^, we could identify a peak indicating the presence of parallel β-sheet in all of the samples. Interestingly, both Aβ_42_-Zn(II) samples (± enrichment) had less compacted levels of parallel β-sheet as compared to fibrils, which we suspect is the reason the absorbance peak migrates to higher wavelengths in the latter case (Figure 5B).

Importantly, the spectrum observed for Aβ_42_-Zn(II) samples matched the one reported previously for Zn(II)-stabilized Aβ40 oligomers,^23^ which reassured that the presence of both parallel and antiparallel β-sheet structures and the absence of α-helix bends are features of this class of Aβ oligomers. Variations in the peak intensities between enriched and nonenriched samples indicate that Zn(II) enrichment might be necessary to maintain the oligomeric structure. Conversely, the removal of Zn(II) following resuspension could lead to the ions dissociating from the aggregates, causing them to lose their ordered structures and adopt more disordered configurations. This structural change, which is also evident in their differing tinctorial properties, could account for the decreased stability of the nonenriched Aβ_42_-Zn(II) samples when incubated at 37 °C.

We further compared the far-UV circular dichroism (CD) spectrum of both enriched and nonenriched Aβ_42_-Zn(II) species with the one of Aβ_42_ fibrils (Figure S2). Aβ_42_ fibrils presented a pronounced negative peak around 218 nm (Figure S2A). This negative ellipticity is characteristic of aggregates rich in β-sheet secondary structures. In contrast, that peak was less defined for Aβ_42_-Zn(II) samples, despite their competence to bind to ThT (Figure S2B).

#### Dot Blotting of Zn(II)-Stabilized Aβ_42_ Oligomers

We also explored the specific reactivity of the control, fibrils, and Aβ_42_-Zn(II) species against three different antibodies: 6E10 (Aβ-sequence-specific antibody), A11 (oligomer-specific antibody), and OC (traditionally defined as an amyloid fibril-specific antibody).^57,58^ Recent evidence demonstrates, however, that both A11 and OC antibodies are able to bind and discriminate between prefibrillar (A11^+^) and fibrillar (OC^+^) oligomeric species. We observed that our Aβ_42_-Zn(II) samples showed reactivity against OC but not A11, consistently to the previously reported Aβ_40_-Zn(II) oligomers^23^ (Figure 5C).

Remarkably, both the control and fibril samples exhibited the same antibody binding profile. As shown in Figure 5D, the levels of OC^+^ signal normalized to the total protein concentration of each dot-blot spot (levels of 6E10 signal) were lower for the control sample and higher for the sample with fibrils. Peptides incubated with zinc displayed intermediate levels of structures capable of binding to the OC antibody. These results are consistent with their ThT-binding profiles (Figure 5E). Altogether, these observations suggest that the presence or absence of zinc ions in the stabilization reaction does not change the overall conformation of the aggregate species; rather, it triggers structural changes that improve oligomer stability and prevent aggregation.

#### TEM of Zn(II)-Stabilized Aβ_42_ Oligomers

**Figure 6:**
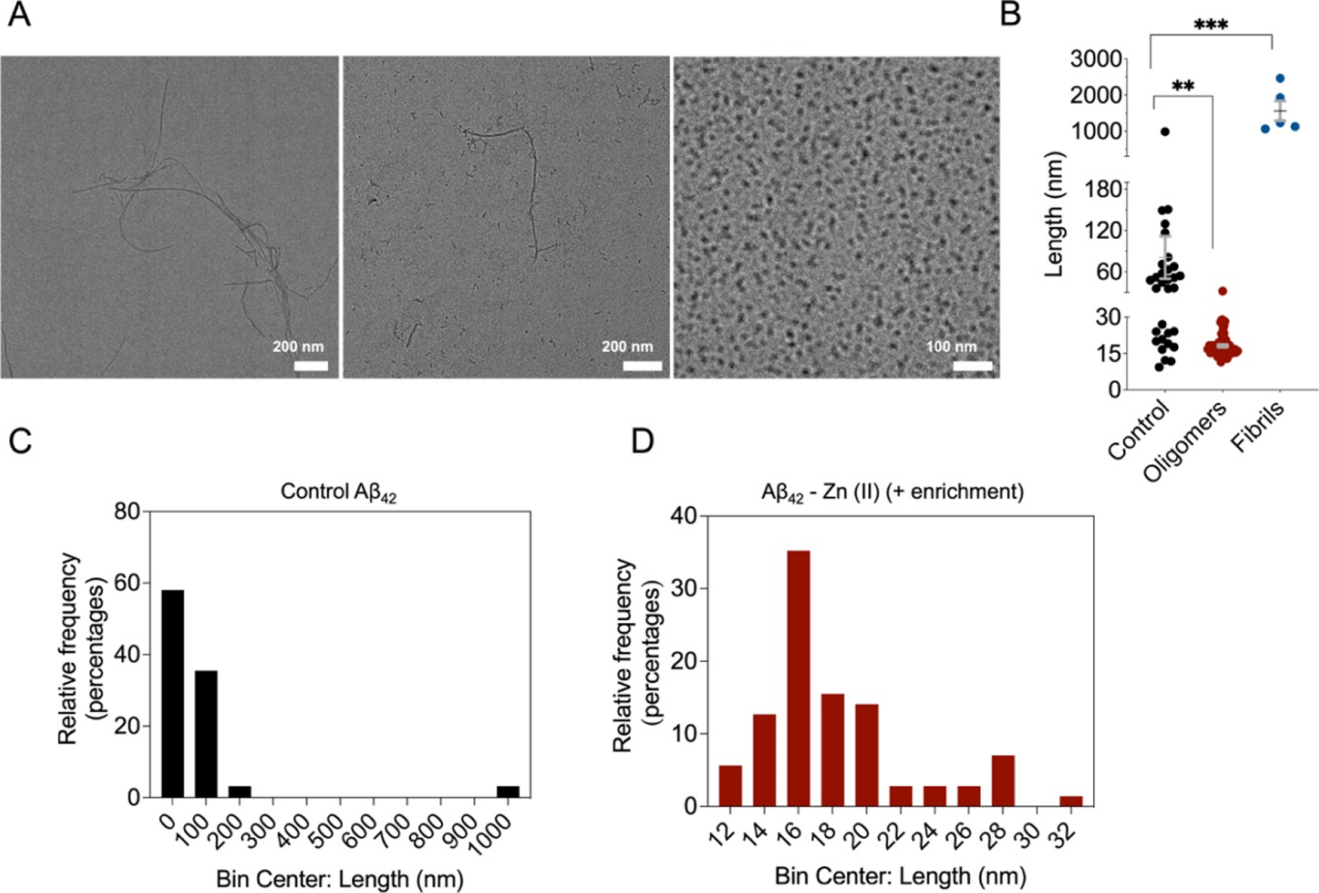
TEM images of Aβ_42_-derived fibrils, control Aβ_42_, and Aβ_42_-Zn(II) samples. (A) Representative images of Aβ_42_ fibrils (left panel), control Aβ_42_ (middle panel), and enriched Aβ_42_-Zn(II) (right panel) products after 20 h of stabilization reaction at 20 °C. (B–D) Control sample is composed of high heterogeneous protein populations (B, C), while enriched Zn(II) samples show greater homogeneity in terms of size and morphology (B,D). Statistical analysis was performed by a one-way ANOVA, applying Bonferroni correction for multiple comparisons. **p-value < 0.01 and ***p-value < 0.001.

Finally, we assessed the size, homogeneity, and morphology of the generated peptide species at the single-molecule level. For this purpose, TEM grids were prepared with: (i) fibrils, (ii) the supernatant fraction of control Aβ_42_ samples, and (iii) the pellet-fraction of enriched Aβ_42_-Zn(II) samples (Figure 6). When the stabilization reaction occurred in the absence of Zn(II) (control reaction), we obtained a heterogeneous population of Aβ_42_ aggregates with assorted sizes (ranging from 15 nm up to 1 μm) and architectures, resembling mostly to protofibrils and small fibrils (Figure 6B,C). In contrast, enriched Aβ_42_-Zn(II) reaction products contained small, highly homogeneous, spherical oligomeric species, of sizes ranging from 12 to 30 nm (Figure 6B,D). Importantly, no amyloid fibrils were detected throughout the grid surface of the prepared samples under the latter condition.

### Cytotoxic Properties of Zn(II)-Stabilized Aβ_42_ Oligomers

We next aimed to assess the cytotoxicity properties of both the control and enriched Aβ_42_-Zn(II) oligomeric species. Most studies support that, upon their intracellular generation and exocytosis, Aβ oligomers interact with a variety of receptors located in the membrane of neurons, from glutamate receptors and complexes such as NMDA-R or mGluR5/PrP^C^ to other lipid or proteoglycan-binding proteins such as LRP.^59,60^ These interactions trigger molecular cascades that lead to mitochondrial dysfunction, decreased ATP levels, increase in reactive oxygen species (ROS) production and calcium influx, ER stress, activation of kinases leading to tau hyperphosphorylation, DNA damage, and, consequently, an irreversible cell death.

**Figure 7:**
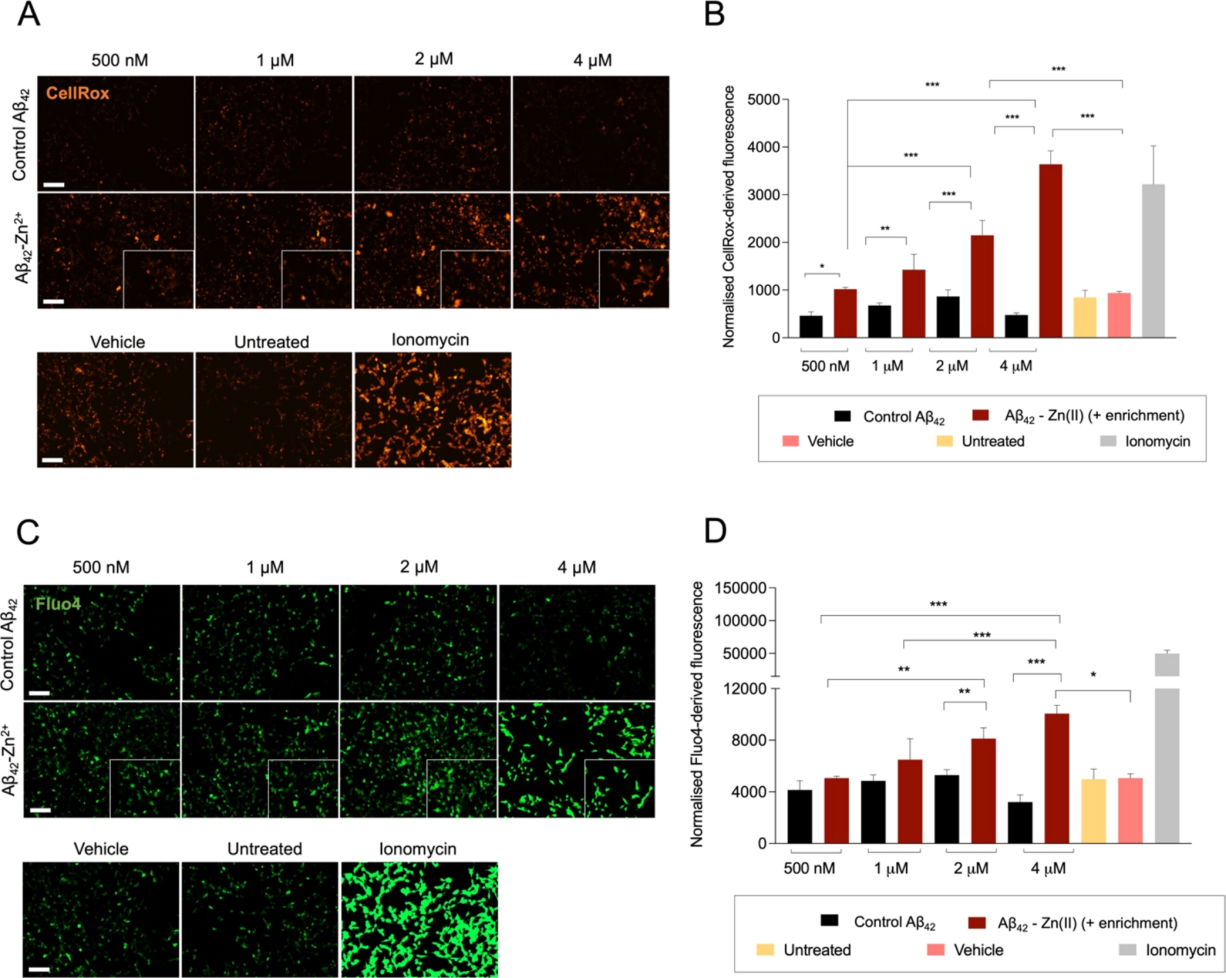
Cytotoxicity assessment of control and Aβ_42_-Zn(II) species. (A) Representative pictures of ROS signal (CellRox) in SH-SY5Y cells treated for 1 h with 500 nM, 1 μM, 2 μM, and 4 μM control Aβ_42_ and enriched Aβ_42_-Zn(II) species generated after a 20 h stabilization reaction at 20 °C. Scale bar = 100 μm. (B) Quantification of ROS production by treated cells. The CellRox-derived fluorescence (read in the RFP channel) was normalized by the total amount of cells (bright field area). Error bars represent standard deviation from three technical replicates. Statistical analysis was performed by a two-way ANOVA, applying Bonferroni correction for multiple comparisons. Ionomycin was excluded from statistical analysis. *p-value < 0.1, **p-value < 0.01, and ***p-value < 0.001. (C) Representative pictures of intracellular calcium levels (Fluo4) of SH-SY5Y cells treated for 1 h with 500 nM, 1 μM, 2 μM, and 4 μM control Aβ_42_ and enriched Aβ_42_-Zn(II) species generated after a 20 h stabilization reaction at 20 °C. Scale bar = 100 μm. (D) Quantification of intracellular calcium levels of treated cells. The Fluo4-derived fluorescence (read in the GFP channel) was normalized by the total amount of cells (bright field area). Error bars represent standard deviation from three technical replicates. Statistical analysis was performed by a two-way ANOVA, applying Bonferroni correction for multiple comparisons. Ionomycin was excluded from statistical analysis. *p-value < 0.1, **p-value < 0.01, ***p-value < 0.001. Vehicle corresponds to buffer enriched with Zn(II).

We examined several of the main hallmarks of Aβ oligomer-derived cytotoxicity in the human neuroblastoma cell line SH-SY5Y, after short-term and long-term exposure of Aβ_42_ aggregates generated in the absence of Zn(II) and Aβ_42_-Zn(II) oligomers generated following the protocol described in this paper. SH-SY5Y cells were treated with increasing concentrations (500 nM, 1 μM, 2 μM and 4 μM) of either control or Aβ_42_-Zn(II) oligomers for 1 h. Cells were then stained with the CellRox dye for the quantification of intracellular reactive oxygen species (ROS) (Figure 7A,B) and the Fluo4 dye for the quantification of intracellular calcium levels (Figure 7C,D). Notably, we observed a dose-dependent increase in the levels of both ROS and calcium influx in cells treated with stabilized Aβ_42_-Zn(II) species, a response that was absent in cells treated with control Aβ_42_ reaction products. Ionomycin, a calcium ionophore, was used as positive control to trigger an increase in intracellular calcium levels and concomitant increase in intracellular ROS. Oligomer-derived toxicity was significantly different from the one observed in vehicle-treated cells when applying concentrations above 1 μM for ROS production (*p*-value < 0.001) and above 2 μM for calcium influx (*p*-value < 0.05).

These results proved that (i) the stabilized Aβ_42_-Zn(II) oligomers were able to trigger AD-relevant cellular toxicity profiles and (ii) the species should be preserved during the treatment, preparation, and delivery, and hence their cytotoxic effect could be measured.

ROS production and calcium influx are closely linked with mitochondrial dysfunction and endoplasmic reticulum (ER) stress in cellular physiology.^60−62^ Mitochondria can generate ATP through oxidative phosphorylation, a process that generates ROS as byproducts.^63^ It is known that ROS production increases with increasing levels of free cytoplasmic calcium, as observed in our cytotoxicity assays (Figure 7). Upon an excessive generation of ROS, critical components of mitochondrial respiratory chain can get damaged, thereby decreasing cellular ATP levels and causing electron leakage that further exacerbates oxidative stress and mitochondrial dysfunction.^63^ In addition to impairing mitochondrial health, Aβ oligomers have also been reported to alter the activity of ER membrane proteins such as calcium-ATPase (SERCA) pumps that actively transport calcium into the ER,^64^ which may contribute to the pronounced calcium influx observed in our assays.

Since our oligomers triggered a cellular response involving an increase in intracellular ROS and calcium levels, we then explored whether a consequent effect on mitochondrial function could be detected in SH-SY5Y cells upon long-term exposure of Aβ_42_-Zn(II) oligomers. As shown in Figure S3, cells treated with Aβ_42_-Zn(II) oligomers showed 50% less reduced MTT levels as compared to the vehicle-treated cells (Figure S3C), presumably due to the impairment of NAD(P)H-dependent cellular oxidoreductase enzymes. Interestingly, treatment with Aβ_42_-Zn(II) samples also led to a decrease in the expression levels of key regulators of mitochondrial quality control and ER stress, such as *PINK1* and *HERPUD2* (Figure S3A).

These observations underscore that our protocol produces oligomeric species with defined biophysical properties, which exhibit a distinct cellular toxicity profile compared with the products formed through aggregation reactions conducted in the absence of Zn(II).

## Discussion and Perspectives

Aβ oligomers are complex and heterogeneous structures of high relevance in AD pathogenesis. Due to the known link between structure and cytotoxic effects of protein aggregates,^65^ it is important to develop techniques that enable the characterization of the whole spectrum of Aβ oligomeric species present in AD brains. This characterization will enable unravelling the structural determinants of Aβ oligomer cytotoxicity and will lay the groundwork for the design of drugs targeting these species. To reach this goal, off-pathway Aβ oligomers need to be isolated for further characterization. This is challenging, given the intrinsic aggregation propensity of Aβ, and the metastable nature of Aβ aggregates.^66−68^

In this context, we built on previous observations about the ability of Aβ to interact with metal ions. These interactions modulate Aβ aggregation and, in some cases, promote the generation of off-pathway oligomers. The half-life of off-pathway Aβ aggregates was reported as longer than their on-pathway counterparts,^69,70^ thus expanding the time window to exert their toxic effects on brain cells. This implies that brain Aβ oligomers stabilized by metal ions may be (i) representative of physiologically relevant species present in AD brains and (ii) responsible of main oligomer-derived cytotoxic processes.

Previous research has utilized Zn(II)^23^ and the metabolite DOPAL^24^ to stabilize oligomeric forms of Aβ_40_. Those studies are of great significance, as they not only provided research tools (i.e., Aβ_40_-Zn(II) or Aβ_40_-DOPAL oligomers) but also standardized protocols for their preparation. It is well-known that studies involving Aβ are technically challenging due to the high tendency of this peptide to misfold into metastable structures of heterogeneous features. Indeed, both the properties and mechanisms of aggregation of Aβ species differ depending on numerous experimental factors, from the purification protocol and buffer composition to the temperature or even the reservoir employed for the storage, aggregation, or manipulation of the peptide. This extreme variability underscores the need for clear, robust methods for the generation of stable Aβ oligomeric structures, with known physicochemical and cytotoxicity properties, thereby promoting their availability to the research community. In this study, we contribute toward this goal by reporting a detailed protocol for stabilizing a homogeneous population of off-pathway Aβ_42_ oligomers using Zn(II).

Previous authors have contributed with valuable characterizations of the effect of Zn(II) on Aβ_42_ oligomerization and fibrillization. In a study published in 2013 by Sharma and co-workers,^43^ the authors aggregated 25 μM monomeric Aβ_42_ with an equivalent concentration of Zn(II) at room temperature for 4 days and observed that Zn(II) both triggers the presence of small Aβ_42_ oligomers concomitantly with insoluble amorphous aggregates. Similarly, another study reported that coaggregation of Zn(II) and Aβ_42_ at a 3:1 molar ratio led to the generation of both globular and fibrillar aggregates.^44^ A more recent publication reported the generation of a heterogeneous population of Aβ_42_-Zn(II) oligomers when incubating 40 μM monomeric peptide with equivalent amounts of Zn(II) for several days, demonstrating that the presence of Zn(II) could disrupt both lag phase and elongation phase of the Aβ aggregation, preventing the oligomers from converting into mature fibrillar structures.^35^ Nonetheless, the authors did not characterize the stability of the generated species, nor did they correlate their physicochemical properties with their toxicity in cellular systems.

Taken together, these studies show that Zn(II) can drive the generation of off-pathway oligomeric aggregates of Aβ_42_ and also highlights the need of an optimized experimental strategy to reach homogeneity and stability of the generated oligomer population, preventing their conversion into amorphous or fibrillar structures. This goal is challenging since the range of conformational states of Aβ_42_ oligomers with Zn(II) is suggested to be broad, with different concentrations of zinc ions changing the aggregation landscape of the peptide.^45^

Here, we reported a step-by-step protocol for the preparation of Zn(II)-stabilized Aβ_42_ oligomers. This protocol generates small Aβ_42_ oligomers, ranging in size from 10 to 30 nm, highly hydrophobic, and ThT-positive with a cross-β structure. Their structure encompasses both parallel and antiparallel β-sheet motifs with an immunoreactivity profile positive for OC and negative for A11. Remarkably, this feature is shared with Aβ oligomers isolated from murine models and postmortem human brain samples, or generated via *in vitro* aggregation techniques.^58,71,72^ Reports investigating OC-positive fibrillar oligomers isolated from human brain extracts argue for their primary role in AD-related cognitive impairment.^72^ Our stabilized Aβ_42_ oligomers also retained cytotoxic characteristics, suggesting that they could be representative of AD brain-relevant species. These Zn(II)-Aβ_42_ oligomers showed alternative features compared to the ones generated by a different study in 2018.^75^ Despite showing the same size ranges and high hydrophobicity levels, these Zn(II) oligomers presented weak immunoreactivity for both A11 and OC antibodies, lower enrichment in the β-sheet content, and a much weaker cytotoxic profile, with concentrations ranging from 10 to 40 μM required to observe phenotypic response in cell cultures.

The approach of using metal ions to stabilize oligomeric species of amyloidogenic proteins extends beyond Aβ. For example, the human islet amyloid polypeptide (hIAPP), whose aggregation has been linked to the death of pancreatic β cells in diabetes, forms oligomers in the presence of Zn(II).^76^ Copper ions can stabilize annular α-synuclein oligomers.^77^ Zn(II) also appears to modulate the amyloid aggregation of TDP-43.^78^ In this paper, we aimed to propose not only a detailed protocol for obtaining Zn(II)-Aβ_42_ oligomers but also a workflow that can be extrapolated to other relevant aggregation-prone peptides and proteins involved in a range of disorders beyond Alzheimer′s disease, such as type 2 diabetes, Parkinson′s disease, and amyotrophic lateral sclerosis (ALS).

Nevertheless, some areas warrant further exploration. While our Zn(II)-Aβ_42_ oligomers are stable when incubated at physiological temperature (37 °C) under *in vitro* conditions, little is known about the stability of these oligomers within a cellular environment. Since alterations of their properties may occur when introduced into biological systems, one needs to be cautious when correlating their structural and cytotoxic profiles. In this regard, the exploration of alternative single-molecule biophysical techniques will be informative. These techniques will enable the estimation of the properties of oligomers within cells, thereby enhancing our understanding of their structure and distribution in complex environments.

TEM is also a valuable tool for visualizing amyloid fibrils.^73^ However, obtaining a good signal-to-noise ratio to observe small oligomers (<10–50 nm) can be challenging. Despite the fact that our TEM images of Aβ oligomers resemble those previously reported,^73,75^ additional nanoscale microscopy techniques (i.e., STORM, PALM, or DNA-PAINT) could be employed to obtain accurate estimates of oligomer size and morphology.^12^ Also, beyond structural assessment, these techniques enable quantification of the real concentration of stable oligomers generated by our protocol. Aggregate concentrations reported in this paper are estimated as relative to the initial monomer concentration before each aggregation or stabilization reaction (monomer equivalents). Additional structural characterization using solid-state nuclear magnetic resonance spectroscopy and cryogenic electron microscopy could be carried out on these oligomers, thanks to their stability and relative homogeneity.

Considering these findings, we conclude that this and previous reports provide experimental procedures for the generation of a panel of stable Aβ_40_ and Aβ_42_ oligomers. By manipulating key parameters in the stabilization reaction, such as using alternative metal ions or metabolites or changing reaction buffer composition and temperature, Aβ oligomeric species with a range of different biophysical and cytotoxic profiles that may resemble other AD-relevant species can be generated. Hence, the exploitation of the versatility of our protocol will help gain a deeper insight into the nature of Aβ oligomers and provide a tool for therapeutic developments targeting these species.

## Materials and Methods

### Preparation of Aβ_42_ Monomers and Fibrils

Recombinant Aβ_42_(M1–42) (MDAEFRHDSGYEVHHQKLVFFAEDVGSNKGAIIGLMVGG VVIA) was expressed and purified and monomers were prepared as previously reported.^74^ Since the binding of Zn(II) has been reported to involve the three histidine residues of Aβ,^75^ the presence of a methionine residue at the N-terminus is not expected to affect the binding of Zn(II) and Aβ_42_. For the generation of Aβ_42_ fibrils, freshly purified monomeric protein was filtered using a 0.22 μm low protein binding filter unit (#SLGV004SL, Millex-GV) in batches of 500 μL of 20 μM concentration. Filtered monomeric peptides were kept in 2 mL LoBind Tubes (#0030108450, Eppendorf) at 37 °C for 72 h without shaking. ThT-binding assay was performed for every batch to assess the reproducibility of the average cross-β content of the fibrillar species.

### Preparation of Aβ_42_ Monomers for Generating Zn(II)-Stabilized Aβ_42_ Oligomers

Lyophilized samples of Aβ_42_ were dissolved in 6 M guanidine hydrochloride (GuHCl) in 50 mM ammonium acetate buffer, pH = 8.5. The solution was kept in ice for at least 1 h. Monomers were separated from oligomeric species and salts using size exclusion chromatography (Superdex 75 10/300 GL column, GE Healthcare, Chicago, IL). Purification was carried out at a flow rate of 0.7 mL/min, using 50 mM ammonium acetate buffer, pH 8 as elution buffer. Aβ_42_ samples were lyophilized in 0.1 mg aliquots and stored at −80 °C.

### Generation of Zn(II)-Stabilized Aβ_42_ Oligomers

0.1 mg aliquots of lyophilized Aβ_42_ monomer were thawed at room temperature for 5 min. Hexafluoro-2-propanol (HFIP, #52517 Sigma-Aldrich) was added to each LoBind tube for peptide solubilization. The protein solution was then sonicated for 10 min at room temperature. The organic solvent mixture was kept at 4 °C overnight to ensure full monomerization of the Aβ_42_ peptides. HFiP was gently evaporated with the use of a N_2_ gas source. Peptides were carefully resuspended with dimethyl sulfoxide (No. 276855, Sigma-Aldrich) by scratching the dried protein from the tube walls with the use of a small pipet tip. Protein solution was then spinned down, sonicated for 10 min at room temperature, and centrifuged at 13 000 rpm for 3 min at 20 °C. The supernatant was transferred to a fresh LoBind Tube (#0030108450, Eppendorf). Monomeric Aβ_42_ in DMSO was diluted to a final concentration of 30 μM in 20 mM Tris hydrochloride (#10812846001, Sigma-Aldrich), pH = 7.4, in a final volume of 370 μL. For the generation of oligomers, ZnCl_2_ ions were added keeping a 1:10 molar excess. Control and oligomer reactions were each incubated at 20 °C for 20 h. Afterward, samples were centrifuged at 13 000 rpm for 30 min at 20 °C. Supernatants were collected in LoBind tubes, and pellet fractions were resuspended with 20 mM Tris hydrochloride buffer (pH = 7.4), enriched with 60 μM ZnCl_2_. Control and oligomer samples were kept at room temperature for all of the subsequent biophysical and cell toxicity analysis.

### SDS-PAGE

Protein samples were first denatured by incubating with the reducing agent NuPAGE LDS Sample Buffer (#NP0007, ThermoFisher) for 5–10 min at 90 °C. Denatured peptides were subjected to an electrophoresis-based separation in NuPAGE Bis–Tris precasted gels with a 4–12% polyacrylamide gradient (#NP0321PK2, ThermoFisher). Running parameters were set for 35 min at a constant 200 V. Gels were stained for 30 min using InstantBlue Coomassie Protein Stain (#ab119211, Abcam) and destained for an additional 30 min using distilled water.

### ANS Binding Assays

8-Anilino-1-naphthalenesulfonic acid (ANS) stocks were prepared in MilliQ H_2_O (#A1028, Sigma-Aldrich). Control or Aβ_42_-Zn(II) samples were diluted to a final concentration of 7 μM in 20 mM Tris hydrochloride buffer, pH = 7.4, enriched with 60 μM ZnCl_2_ when appropriate, using protein LoBind tubes. ANS solution was added to each tube for a final concentration of 21 μM. Samples were then distributed in 80 μL aliquots into 3881 Corning plates. End-point and kinetic ANS assay were carried out at 37 °C. ANS-derived emission spectra (λ_exc_ = 380 nm) were captured using a plate reader (BMG Labtech, Aylesbury, U.K.).

### Turbidity

Turbidimetry of the protein samples as an indirect measure of their average size was assessed by measuring the absorbance of the samples at 500 nm. Blank subtracted absorbance at λ = 500 nm was reported.

### ThT-Binding Assays

Thioflavin T (ThT) stocks were prepared in MiliQ H_2_O (#T3516, Sigma-Aldrich). Control or Zn(II)-treated Aβ_42_ samples were diluted to a final concentration of 4 μM in 20 mM Tris hydrochloride buffer, pH = 7.4, enriched with 60 μM ZnCl_2_ when appropriate. Protein LoBind tubes were used for all of the preparations. ThT solution was added to each tube for a final concentration of 40 μM. Samples were then distributed in 80 μL aliquots into a 96-well half-area, low-binding, clear bottomed, and PEG coated plate (#3881, Corning). End point ThT assay was carried out at 25 °C, while kinetic assays were performed at 37 °C. ThT fluorescence (λ_exc_ = 440 nm; λ_em_ = 480 nm) was traced with the use of a plate reader (BMG Labtech, Aylesbury, U.K.).

### Fourier Transform Infrared Spectroscopy

Control, Aβ_42_-Zn(II), and fibril samples were centrifuged at 13 000 rpm for 30 min. Supernatants were discarded, and pellets were resuspended in 10 μL of 20 mM Tris hydrochloride pH = 7.4, enriched with 60 μM ZnCl_2_ whenever appropriate, obtaining a final concentration of protein equivalent to 1.05 mM. ATR-FTIR spectroscopy was carried out using a Bruker Verter 70 spectrometer equipped with a diamond ATR element (Bruker, Billerica, MA). Spectra were acquired with a resolution of 4 cm^–1^ and processed by means of Origin Pro software. For example, two spectra were averaged (each spectrum obtained from 128 scans), and subsequently, the second derivative was calculated applying the Savitzky–Golay filter (second order, 12 points).

### Dot-Blot Assay

After 20 h of stabilization reaction, both control and Aβ_42_-Zn(II)-enriched supernatant and pellet fractions were diluted at a 1:2 ratio in the appropriate buffer (20 mM Tris hydrochloride, pH = 7.4 with or without enrichment with 60 μM ZnCl_2_). Two μL of each sample was spotted on a nitrocellulose membrane of 0.2 μm pore size. To avoid nonspecific binding of antibodies, membranes were blocked by incubating with 5% nonfat dry milk in 0.1% v/v Tween-20 PBS (PBS-T) solution for 1 h at room temperature. Afterward, membranes were incubated overnight with 1:1000 6E10 antibody (#MABN10, Sigma-Aldrich), 1:1000 OC (#AB2286, Sigma-Aldrich) or 1:750 A11 (#AHB0052, Invitrogen) primary antibodies diluted in a blocking solution. Membranes were then washed in 0.1% v/v Tween-20 PBS (PBS-T) solution three times prior to their incubation with the appropriate Alexa^488^-conjugated secondary antibody at a 1:5000 dilution in blocking solution for 1 h at room temperature. Secondary antibody was washed three times. Detection of the desired protein was carried out by measuring AlexaFluor^488^-derived fluorescence in a ChemiDoc Imaging System (BioRad, UK).

### TEM Sample Preparation

3 μL of control, Aβ_42_-Zn(II), and fibril Aβ_42_ samples, diluted to a final concentration of 15 μM (monomer equivalents) were spotted over the coated side of copper-based TEM grids. Samples were incubated for 3 min while covered by a Petri dish lid to avoid any contamination from dust or air particles. Subsequently, samples were gently wicked-off using a Whatman filter paper, avoiding touching the main surface of the grid. Three μL of uranyl acetate (2% w/v) was added to the treated grid surface and incubated for 1–2 min. Uranyl acetate was wicked-off, and grids were dried at room temperature for 5 min under an appropriate cover protection. Grids were stored in suitable boxes at room temperature. Imaging of fixed and stained proteins was performed using an electron microscope (Thermo Scientific (FEI) Talos F200X G2 TEM, Chemistry Department, University of Cambridge). Quantification of images was performed using ImageJ software (NIH, Bethesda, MD).

### Culturing Human Neuroblastoma SH-SY5Y

The immortalized human neuroblastoma cell line (SH-SY5Y) was grown in Dulbecco’s Modified Eagle’s Medium (DMEM) combined at a 1:1 ratio with Ham’s F-12, supplemented with (1) GlutaMAX (#10565018, Gibco) for promoting better cell viability and growth levels and (2) 10% v/v heat-inactivated fetal bovine serum (iFBS) (#10082147, Gibco) to provide the essential protein, lipids, and growth factors lacking in the basal medium. Cells were grown as a monolayer in noncoated culture flasks and maintained at 37 °C under an atmosphere containing 5% CO_2_.

### ROS Production and Calcium Influx in SH-SY5Y

SH-SY5Y cells were plated in 96-well TC-treated plates (#CLS3595, Corning) at a cell density of 10 000 cells per well. Cultures were kept at 37 °C for 24 h prior to treatment. For treatment preparation, the cell culture medium was removed from each well and replaced by 50 μL of a 2× solution of CellRox (#C10493, ThermoFisher), Fluo4 (#F14201, ThermoFisher), and Hoechst 33342 (#H3570, ThermoFisher) dyes in Live Cell Imaging Medium (LCIM, #A14291DJ, Invitrogen) supplemented with an antibiotic/antimycotic solution (100 U/mL of penicillin, 100 μg/mL of streptomycin, and 0.25 μg/mL of amphotericin B, #15240096, Gibco). In parallel, an intermediate 2× stock of the corresponding control or Aβ_42_-Zn(II) samples was prepared in LCIM. 50 μL of each sample was added to the supernatant of the corresponding wells to reach the desired final 1× concentration. The cells were incubated with the appropriate treatment and dyes for 30 min at 37 °C prior to image acquisition under the Cell Imaging Multi-Mode Reader Cytation 5 (BioTek, U.K.). Image analysis was performed using Gen5 software (BioTek, UK).

## Supporting Information Available

Table detailing the fold change in cross-β content from the ThT aggregation kinetics of the stabilization reaction products (Table S1); stoichiometric effects of copper and DOPAL on the aggregation of Aβ_42_ (Figure S1); comparison of the far-UV CD spectra of nonenriched and enriched Aβ_42_-Zn(II) samples with Aβ_42_ fibrils (Figure S2); results on the impact of Aβ_42_-Zn(II) samples on mitochondrial function (Figure S3); and materials and methods for the CD, MTT, and qPCR assays (PDF)

## Abbreviations

AD: Alzheimer’s disease
Aβ: amyloid-β peptide
Aβ42: 42-residue form of Aβ
Zn(II): zinc ion with a +2 oxidation state
