# Supplementary material for: Preparation and Characterization of Zn(II)-Stabilized Aβ42 Oligomers
Source: ACS Chem Neurosci. 2024 Jul 9;15(14):2586–99. doi: 10.1021/acschemneuro.4c00084 (PMC11258685; doi:10.1021/acschemneuro.4c00084)
Supplement: Supplementary file 1 — cn4c00084_si_001.pdf [file cn4c00084_si_001.pdf]

SUPPLEMENTARY INFORMATION

Preparation and Characterization of Zn(II)-Stabilised Aβ$_{42}$ Oligomers

Alicia González Díaz[1], Rodrigo Cataldi[1], Benedetta Mannini[1,2], Michele Vendruscolo[*1]

[1]*Centre for Misfolding Diseases, Yusuf Hamied Department of Chemistry,*
University of Cambridge, Cambridge CB2 1EW, United Kingdom
[2]*Department of Experimental and Clinical Biomedical Sciences Mario Serio,*
University of Florence, 50134, Italy

[*]To whom correspondence may be addressed: mv245@cam.ac.uk

Far-UV circular dichroism

Circular dichroism (CD) spectra for Aβ-Zn(II) samples (with and without enrichment) and fibrillar Aβ42 were collected with a J-810 spectropolarimeter (Jasco, Tokyo, Japan), using a 1 mm quartz cuvette and a temperature-controlled Peltier holder set to 20 °C. We recorded the spectra by averaging 10 to 30 scans, across a wavelength range of 200-250 nm, with a scan rate of 50 nm/min and a resolution of 1 nm. For all samples, CD signal was corrected by subtracting the CD signal from the buffer. The resultant spectra were smoothed using the GraphPad software, by averaging 13 neighboring points and using a 4th order smoothing polynomial.

Assessing MTT reduction of SH-SY5Y cells treated with Zn(II)-stabilized $A\beta_{42}$ oligomers

5 mg/mL stocks of yellow tetrazolium salt (3-(4,5-dimethylthiazol-2-yl)-2,5-diphenyltetrazolium bromide or MTT, #M5655, Sigma Aldrich) were prepared by diluting the molecule in RPMI Medium, without phenol red (#11835030, Gibco). Solution was filtered under sterile conditions and stored in small aliquots at -20 °C for a maximum period of 6 months. Working MTT solution was freshly prepared in sterile RPMI medium, at a final concentration of 0.5 mg/ml. 24 h after incubation with the corresponding $A\beta_{42}$-derived oligomeric species, the full content of the wells was carefully removed and replaced by 100 µL per well of working MTT solution. Cells were then incubated at 37 °C for 4 h. Afterwards, cells were lysed by adding 100 µL per well of the stop solution (20% SDS, 50% N, N-dimethylformamide, pH 4.7), followed by an incubation step of 1 to 2 h, shaking at 550 rpm. Purple formazan absorbance values were measured at a wavelength of 590 nm using a plate reader (BMG Labtech, Aylesbury, UK).

Expression levels of genes involved in ER and mitochondrial function

SH-SY5Y cells were plated at 50% confluency in 12-well plates and treated with 500 nM and 1 µM $A\beta_{42}$-Zn(II) samples. Percentage of FBS was reduced to 1% to reduce the probability of oligomers to interact with the components of the serum. Cells were incubated with the protein treatment for 24h prior mRNA extraction.

(a) Cellular lysis for RNA extraction. Cell medium was carefully removed from the corresponding wells with the use of a 1 mL pipette. Afterwards, 400 µL of lysis buffer (#12183025, Invitrogen) freshly enriched with 1% β-mercaptoethanol were added per well, followed by 5-10 min incubation at room temperature. Full lysates from each individual well were transferred to separate RNAse free DNA LoBind tubes, which were subsequently flash-frozen and stored at -80 °C for further RNA purification. Cell lysates were thawed at room temperature. RNA purification was performed using the PureLink™ RNA Mini Kit as per

manufacturer instructions. RNA purity and yield was further quantified with a NanoDrop spectrophotometer (Thermo Fisher Scientific). Only samples with absorbances ratios of $\lambda_{260/280} \approx 1.8\text{-}2$ and $\lambda_{260/230} \approx 2\text{-}2.2$ were used for cDNA generation and qPCR amplification.

(b) Messenger RNA analysis by quantitative PCR (qRT-PCR). 1 µg of RNA, was used to synthesize cDNA in a 20 µl volume with the High-Capacity RNA-to- cDNA kit (#4388950, Applied Biosystems). The quantitative real time polymerase chain reaction (qRT-PCR) was carried out in a volume of 20 µl with the TaqMan Gene Expression Master Mix (#4369016, Applied Biosystems) and 1X of each specific oligonucleotide (ThermoFisher). An initial 10 min denaturation step of 2 min at 50 °C and 10 min at 95 °C was followed by 40 cycles of denaturation (15 sec at 95 °C), annealing (30 sec at 60 °C) and elongation (30 sec at 60 °C). Both retro-transcription and qPCR reaction took place in a QuantStudio™ 3 Real-Time PCR System (Applied Biosystems, UK). Messenger RNA (mRNA) levels were evaluated with the ΔΔCt method, using *ACTB* or *GAPDH* housekeeping genes, whose expression was determined to be stable between experimental conditions.

Table S1. Fold change of β-sheet content upon aggregation at 37 ºC of 10 min, 4 h, 8 h and 20 h stabilisation reaction products. Mean fold change of β-sheet content after 20 h of aggregation at 37 °C of the supernatant fraction of the control sample (M-Aβ_{42}) and the pellet fractions of enriched and non-enriched Aβ_{42}-Zn(II) samples previously subjected to 10 min, 4 h, 8 h or 20 h of stabilisation reaction at 20 °C.

Stabilisation reaction time points	Fold change of β-sheet content ThT fluorescence intensity ratio ($F(t_0)/F(t_{plateau})$)					
	Control Aβ_{42}		Aβ_{42}-Zn(II)		Aβ_{42}-Zn(II) + enrichment	
	**Mean**	**SD**	**Mean**	**SD**	**Mean**	**SD**
10 min	5.40	0.12	1.78	0.04	1.31	0.03
4 h	6.18	0.10	2.07	0.02	1.59	0.04
8 h	5.81	0.19	2.22	0.09	1.57	0.08
20 h	5.52	0.12	1.95	0.02	1.33	0.01

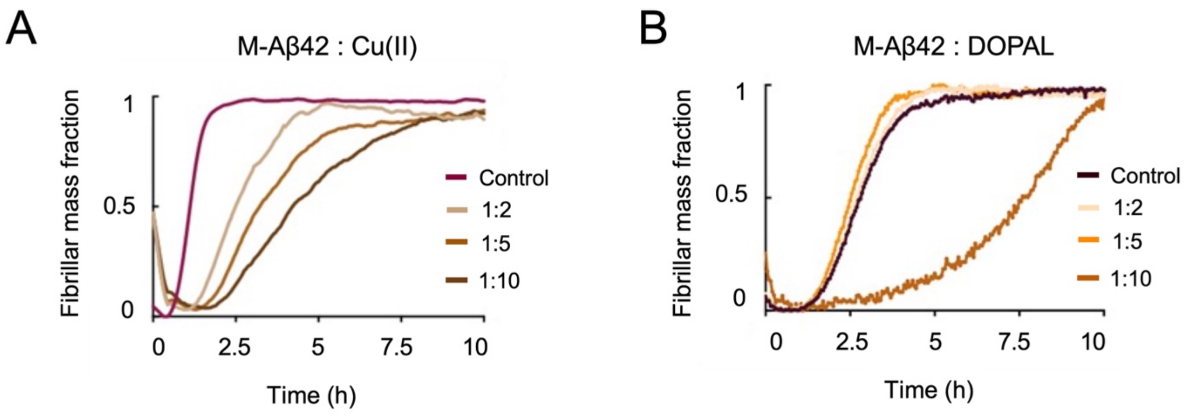

Figure S1. Influence of Cu(II) and DOPAL on the *in vitro* aggregation kinetics of Aβ_{42}. (A-C) ThT fluorescence profiles of the aggregation of 5 μM monomeric Aβ_{42} in the absence or presence of increasing molar equivalents of Cu(II) (**A**) and DOPAL (**B**).

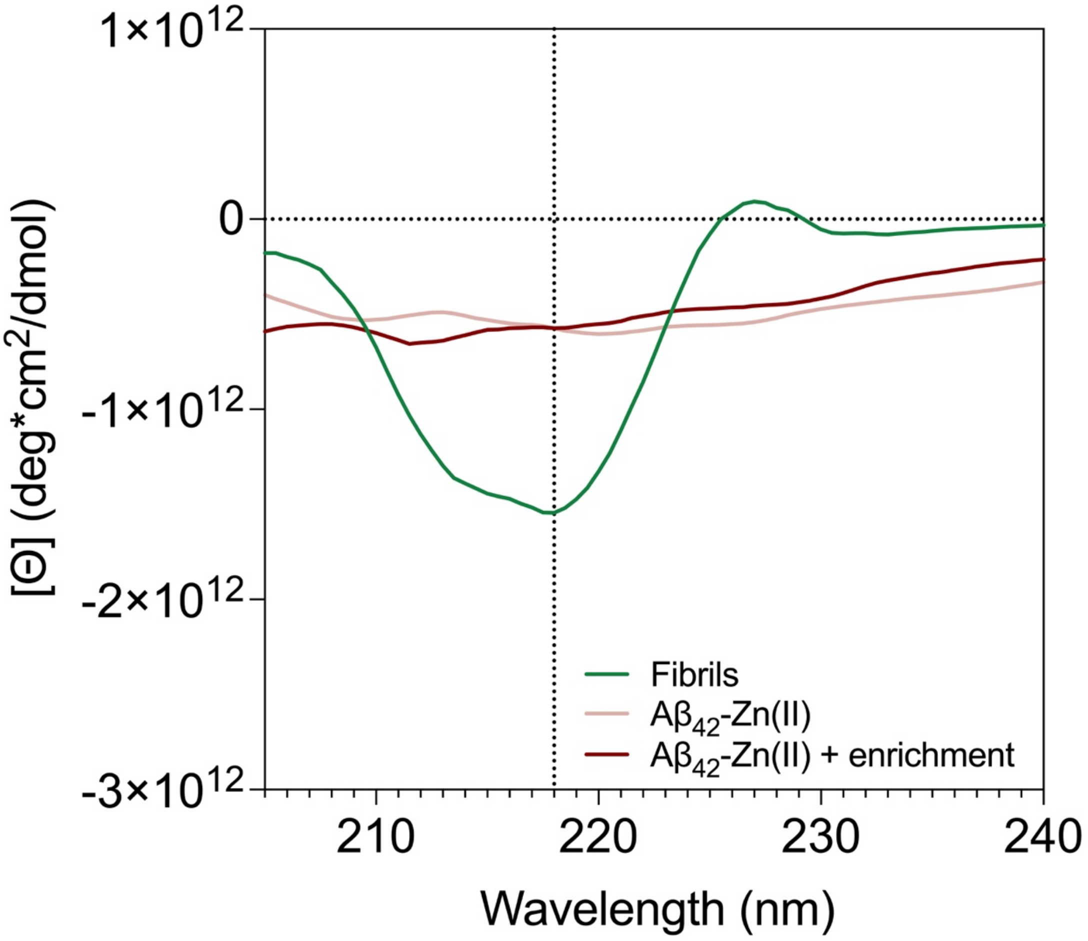

Figure S2. Comparison of the far-UV CD-spectra non-enriched and enriched Aβ_{42}-Zn(II) samples with Aβ_{42} fibrils. Far-UV CD spectrum of Aβ42 aggregated for 24 h at 37 °C (fibrils) or for 20 h at 20 °C following the stabilisation reaction protocol, with our without further Zn(II) enrichment.

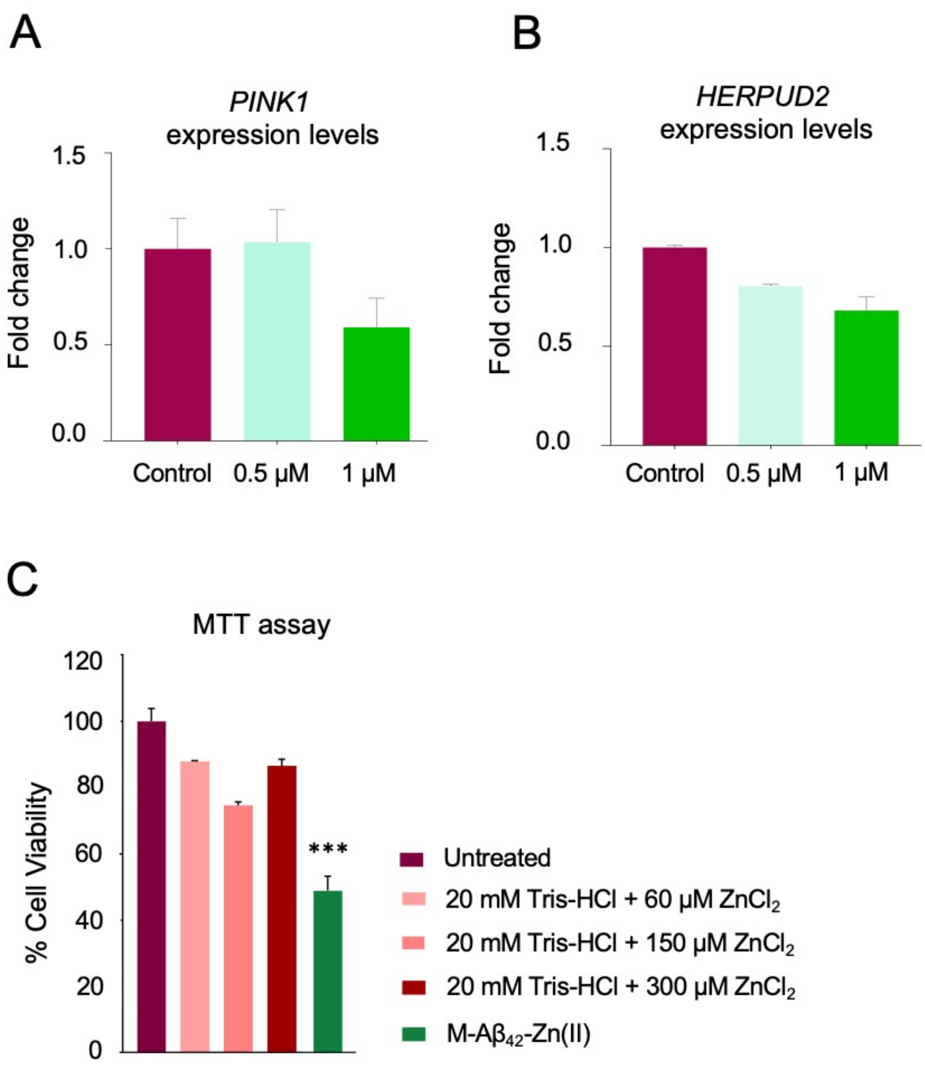

Figure S3. Impairment of mitochondrial function upon treatment with Aβ$_{42}$-Zn(II) samples. (**A**,**B**) mRNA levels of PINK1 (A), a regulator of the degradation of dysfunctional mitochondrial, and HERPUD2 (B), a regulator of ER stress and the unfolded protein response in SH-SY5Y cells treated with increasing concentrations of Aβ$_{42}$-Zn(II) samples previously incubated at 20 °C for 20 h. (**C**) Viability of cells treated with 1 μM Aβ$_{42}$-Zn(II) samples. Error bars represent standard deviation. Statistical analysis was performed by a one-way ANOVA, applying Bonferroni correction for multiple comparisons. p-value ***<0.001.
